# Does a mineral wristband affect balance? A randomized, controlled, double-blind study

**DOI:** 10.1186/s40463-015-0079-1

**Published:** 2015-06-26

**Authors:** Eva Ekvall Hansson, Anders Beckman, Liselott Persson

**Affiliations:** Department of Health Sciences, Health Science Centre, Lund University, Baravägen 3, SE222 41 Lund, Sweden; Department of Clinical Sciences in Malmö, Clinical Research Centre, Lund University, Jan Waldenströmsgata 25, SE205 02 Malmö, Sweden

**Keywords:** Postural control, Balance, Hologram, Force plate

## Abstract

**Background:**

Having good balance is a facilitating factor in the performance of everyday activities. Good balance is also essential in various sport activities in order to both get results and prevent injury. A common measure of balance is postural sway, which can be measured both antero-posteriorly and medio-laterally. There are several companies marketing wristbands whose intended function is to improve balance, strength and flexibility. Randomized controlled trials have shown that wristbands with holograms have no effect on balance but studies on wristbands with minerals seem to be lacking.

**Objective:**

The aim of this study was to investigate if the mineral wristband had any effect on postural sway in a group of healthy individuals.

**Study design:**

Randomized, controlled, double-blind study.

**Material/Methods:**

The study group consisted of 40 healthy persons. Postural sway was measured antero-posteriorly and medio-laterally on a force plate, to compare: the mineral wristband, a placebo wristband, and without any wristband. The measurements were performed for 30 s, in four situations: with open eyes and closed eyes, standing on a firm surface and on foam. Analyses were made with multilevel technique.

**Results:**

The use of wristband with or without minerals did not alter postural sway. Closed eyes and standing on foam both prolonged the dependent measurement, irrespective if it was medio-lateral or antero-posterior. Wearing any wristband (mineral or placebo) gave a small (0,22-0,36 mm/s) but not statistically significant reduction of postural sway compared to not wearing wristband.

**Conclusion:**

This study showed no effect on postural sway by using the mineral wristband, compared with a placebo wristband or no wristband. Wearing any wristband at all (mineral or placebo) gave a small but not statistically significant reduction in postural sway, probably caused by sensory input.

## Introduction

Having good balance is a facilitating factor in the performance of everyday activities [1]. Good balance is also essential in various sport activities in order to both get results and prevent injury [2]. Research about the effect of balance training on sports performance is however inconclusive [3]. Despite this, possibilities to improve balance and thereby possible enhancement of performance are attractive.

Balance has been defined as “Sensing the position of the body’s centre of mass and moving the body to adjust the position of the centre of mass over the base of support provided by the feet”, by Nashner *et al.* [4]. Several systems interact to maintain balance: vision, the somato-sensory system, and the vestibular organ. These systems interact and register inputs from the surroundings, which are integrated and processed in the central nervous system. The vestibulo-ocular reflex (VOR) coordinates eye and head movements, making it possible, for example, to walk and read signs at the same time [5]. Through the head-neck skeletomotor system, the cervico-ocular reflex interacts with the VOR, providing information about head movements in relation to the trunk [6]. Sensory receptors in the skin as well as mechanoreceptors in the muscles provide input as to how gravity affects the body [7, 8]. Input from these different parts of the balance system is constantly reconsidered and a response from the motor cortex is sent back. This means that even when standing still, the body is constantly in motion. This motion is called postural sway [1], and can be measured by using a force plate and measuring the movement of the center of pressure (COP) in the medio-lateral (ML) direction as well as in the antero-posterior (AP) direction [1, 9].

### Wristbands and function

There are several companies that market wristbands aiming to improve functions such as balance, mobility/agility and strength [10–12]. The technology differs: holograms, ions, protons and minerals are claimed to influence function and well-being [10–12]. Therapists and coaches are often asked by patients and athletes about the efficacy of these wristbands and their impact on balance. Randomized controlled trials have shown that wristbands with holograms have no effect on balance [13, 14], but studies on wristbands with minerals seems to be lacking. Mineral wristbands are marketed to be able to dramatically improve balance by simply putting it on [12]. Therefore, it is important to perform studies on mineral wristbands as well, in order to provide therapists and coaches with adequate information about different wristbands, giving them possibilities to give correct advise to patients and athletes.

The aim of this study was therefore to investigate a mineral wristband’s impact on postural sway in a group of healthy individuals, compared to a placebo wristband, and no wristband at all.

## Methods

### Subjects

The study group was 40 healthy volunteers, aged 20 to 69 years (mean 35 ± 17), of whom 25 (62 %) were women. They were 162 to 191 cm tall (mean 176 ± 8.5) with a body mass index 20.3 to 30.5 kg/m^2^ (mean 23.7 ± 2.9). The inclusion criteria were no dizziness or balance problems, no neck pain, no newly acquired injury to the hip, knee, or foot for the last two months, and corrected visual impairment, if any. None of the participants had any hearing problems and none of them had used a wristband before. The subjects were selected among students and staff at Lund University.

### Ethics

Participation in the study was strictly voluntary. All participants gave their informed consent before entering the study. The study was approved by the Regional Ethical Review Board in Lund no 2014/127.

### Procedure

In this randomized double-blind study, the patricipants were tested on a force plate (Good Balance™, Metitur Ltd, Finland, http://www.papapostolou.gr/clientfiles/file/pdf/Good_Balance_Brochure.pdf) wearing the Bionic-sport wristband (The BIONICSPORT™, BionicFamily®, www.bionicband.com), a placebo wristband (without minerals but made of silicon and the same colour, weight and circumference as the mineral wristband), or no wristband. Tests were performed on the force plate with open and closed eyes and standing on 3 cm thick foam on the force plate with open and closed eyes.

All measurements were standardized and the tests were performed in the same room. Before the measurements, each participant received verbal and written information and signed a paper on the voluntary participation and filled in a report on personal data. Since the whole procedure took about 30 min, the participants was offered to drink a glass of water before the measure to reduce the risk of dehydration. The participants removed shoes, jewelry, watches, and electronic equipment such as mobile phones.

On the force plate, the participants were placed with the feet in a standardized position with 30° of external rotation, marked on the force plate. The neck was positioned in 20° of flexion, and the participants were asked to focus eyes on one spot on the wall, at a distance of 1.5 m, individually adjusted to height and neck position. This is to avoid proprioceptors from the neck to have an impact on the postural wobbling [15]. The participants could not see the force platform’s computer screen during the measurements. The verbal instructions were standardized. The participants were instructed to keep the arms hanging freely and during the test not to speak. Each participant then performed a test measurement for 30 s with eyes open and on a firm surface, in order to familiarize themselves with the plate and the test approach.

The procedure was carried out without wristband, with the placebo wristband and with the mineral wristband. The order in which each of the three procedures was carried out, was randomly selected, using a random list.

The measurements were performed by two test-leaders. The same test-leader gave instructions to the participants, and applied the wristband to be used at each measurement. This test leader had no knowledge of which bracelet was used, or the randomized test scheme. Another test leader provided the first test leader with the wristbands according the random list, and documented the measurements into the computer system Good Balance TM, Metitur Ltd., Finland.

### Equipment

The postural sway was measured by a computerized system, which consisted of a triangular plate with force sensors at each corner. Force plate was connected to a PC where the program Good Balance TM, Metitur Ltd, Finland, was installed and synchronized with the plate. Based on signals from each corner of the force plate, the system analyzed the average speed of the movements based on the COP both ML and AP. These values are indicated in mm/s. The system corrects for any differences in the height and the COM of the various test subjects. The force plate was tested and calibrated prior to use and a basic calibration was implemented automatically every time the computer program was started. There were also automated checks every two hours. This model of force plates has been tested for validity and intrasession- and test-retest reliability [9, 16].

The mineral wristband was made of silicone and, according to the manufacturer, it contains mineralized surgical steel and has the “highest frequency” which therefore makes the greatest impact on balance [12]. The placebo wristband was also made of silicone and looked the same as the mineral wristband.

### Statistical methods

Considering standard deviation for measures on the force plate [9] a power of 80 % and the significance level set at 0.05, a sample size of 30 subjects was required [17]. Since we also wanted to detect small differences between the groups, sample size was set to 40.

Due to the fact that the observations are repeated measures within the same subject, there is dependence between measurements. To correct for this deviation from the pre-requisites for a traditional regression model, we used a multilevel approach, where a correction for dependence is built into the model. [18, 19]. We regarded the repeated measures to be clustered within the subjects, thus giving a two level structure. The dependent variables used were ML and AP. The independent variables used were vision (eyes open or closed), surface (firm surface and foam) and wristband: without, placebo or mineral. Each dependent variable (ML, AP) was analyzed separately with all independent variables.

The multilevel analysis started with a so-called empty model, *i.e.* a model with only a fixed part and a random part. The fixed part models the effect of the mean that underlies all observations. The random part consists of a decomposition of the total variance into two levels: variance between subjects (second level) and between occasions within subjects.

The empty model was then extended by including the independent variables in the fixed part of the model (eyes, surface and wristband). Inclusion was made stepwise, but only the last model with all variables is presented.

Analysis was made with MlWin, v 2.30 [20] Residual (or restricted) maximum likelihood (REML) was used for all analysis. REML estimation takes into account the loss of degrees of freedom resulting from the estimation of the parameters of the fixed part. This has an indirect effect on the estimates of the fixed part.

The results are presented with 95 % confidence intervals [21].

A reduction in the total variance is an indicator of a better “fit” of the model, as is the reduction in the deviance.

## Results

Standing with eyes open on a firm surface caused the smallest postural sway in all dimensions (ML 7,47 mm/s, AP 13,64 mm/s) and standing with eyes closed on foam prolonged the sway (ML +3,82 mm/s, AP +8,12 mm/s). The use of wristband with or without minerals did not alter the sway. The overall mean values and SD for each variable are displayed in Table 1 and each test is displayed in Table 2.

**Table 1:** Mean values and standard deviation (SD) for medio-lateral sway (ML) and anterior-posterior sway (AP)

		ML	AP
		mm/s (SD)	mm/s (SD)
Overall mean		5.41 (2.59)	9.39 (4.49)
Eyes closed		6.58 (2.97)	11.70 (4.90)
Foam standing		6.15 (2.85)	11.20 (4.91)
Wristband	None	5.56 (2.59)	9.58 (4.49)
	Placebo	5.34 (2.68)	9.22 (4.51)
	Mineral	5.32 (2.50)	9.36 (4.50)

**Table 2:** Mean values and standard deviation (SD) for medio-lateral sway (ML), anterior-posterior sway (AP) in relation to proprioception, sight and wristband

Proprio	Eyes	Wristband	ML	AP
			mm/s (SD)	mm/s (SD)
Firm	Open	Without	3.87 (1.16)	6.03 (2.07)
Firm	Shut	Without	5.66 (2.30)	9.35 (3.45)
Soft	Open	Without	4.91 (1.52)	8.78 (2.58)
Soft	Shut	Without	7.81 (3.14)	14.17 (4.90)
Firm	Open	Placebo	3.72 (1.03)	5.72 (1.76)
Firm	Shut	Placebo	5.60 (2.65)	9.18 (3.17)
Soft	Open	Placebo	4.53 (1.15)	8.00 (2.14)
Soft	Shut	Placebo	7.52 (3.41)	13.99 (5.27)
Firm	Open	Mineral	3.63 (1.25)	6.02 (1.64)
Firm	Shut	Mineral	5.53 (2.04)	9.40 (3.45)
Soft	Open	Mineral	4.75 (1.42)	8.03 (2.45)
Soft	Shut	Mineral	7.37 (3.18)	13.98 (5.18)

### Multilevel analysis – fixed part

The sway in AP was almost 50 % higher than the sway in ML (Table 3). However, both measures show similar variations when the independent variables are introduced, *i.e.* a prolonging of sway when eyes are closed and the surface is non-firm (foam). However, the use of a wristband does not significantly alter the sway.

**Table 3:** Means and 95 % confidence intervals (CI) for fixed and random parts and deviance

		ML-speed		AP-speed	
		mm/s (95 % CI)	mm/s (95 % CI)	mm/s (95 % CI)	mm/s (95 % CI)
		Empty model	Full model	Empty model	Full model
Fixed part					
Intercept		5,41 (5,18 - 5,64)	7,47 (6,90 - 8,04)	9,39 (8,60- 10,18)	13,64 (12,76 - 14,52)
Foam			1,48 (1,22 - 1,74)		3,54 (3,14 - 3,94)
Eyes closed			2,34 (2,08 - 2,60)		4,58 (4,18 - 4,98)
Wristband	None		0		0
	Placebo		−0,22 (−0,53 - 0,10)		−0,36 (−0,14 - 0,86)
	Mineral		−0,24 (−0,56 - 0,07)		−0,22 (−0,28 - 0,72)
Random Parts					
Variance					
Within subjects		4,25 (3,70 - 4,80)	2,14 (1,86 - 2,42)	14,6 (12,72 - 16,48)	5,43 (4,72 - 6,14)
Between subjects		2,43 (1,23 - 3,63)	2,61 (1,41 - 3,8)	5,54 (2,64 - 8,44)	6,31 (3,41 - 9,21)
Deviance		2139	1838	2718	2282

### Multilevel analysis – random part

The empty model reveals for all outcomes a similar pattern, *i.e.* both random variance components (between subjects (second level) and between occasions within subjects) differ from zero. This justifies the use of a multilevel model. Similarly, the introduction of independent variables reveals an almost uniform pattern: Closed eyes and standing on foam both prolong the dependent measurement, irrespective if it is ML or AP. This also reduces the size of the random part, as an effect of a better model fit, also observed in the reduction in deviance shown in Table 3.

## Discussion

In this study, the use of a mineral wristband did not affect postural sway, neither compared to wristband whitout minerals nor compared to no wristband at all. Standing with eyes open on a firm surface caused the smallest postural sway in all dimensions and standing with eyes closed on foam prolonged the sway, irrespective of which wristband was used or if no wristband was used. Closed eyes and standing on foam both prolong the dependent measurement, irrespective if it was ML or AP and also irrespective wristband or no wristband.

As shown in other randomized controlled trials, on other types of wristbands, there was no difference between placebo or mineral wristband [13, 14]. These studies were similar to our in design, participants and one of them also used a force plate to measure balance [13].

Light touch of the skin has proven to affect postural sway among persons with balance deficits [22] and persons with poor ability to feel the direction of a tactile sensation can have reduced postural stability [23]. Thus, the small but statistically significant reduction in sway seen when using any of the wristbands in our study can be caused by the sensory information provided through the wristband’s contact with the skin. Another explanation might be the expectation from the participants that without the wristband, balance would be worse. Studies about the placebo effect has shown that positive expectations increases the likelihood of reporting feeling better after surgery [24] and that dopamine receptors can be affected by treatment with placebo [25]. However, our study cannot answer whether the effect of the wristband is accentuated if the wearer believes that the wristband can improve performance.

The present study is a randomized controlled trial, where the same persons performed all the measures, the same protocol was used for all subjects and an independent person performed the randomization. The assessor was also blind to if the wristband used was placebo or mineral.

The subjects in our study only wore the wristband during the measurements. If wearing the wristband during a longer period of time or during sport activity or competition actually gives effect on results is not yet studied. We only measured static balance in this study, further studies is needed on dynamic balance, especially when performing a task. Further studies are also needed on the impact of mineral wristband on strength and flexibility. Also, the placebo effect has to be taken into account when considering the effect of the wristband; a small increase in performance caused by the placebo effect when the athlete is convinced that the wristband will improve performance, is in fact a real improvement.

## Conclusion

Wearing a mineral wristband did not affect postural sway in this group of healthy individuals, compared to a placebo wristband or no wristband at all. Wearing any wristband (mineral or placebo) gave a small but not statistically significant reduction of postural sway, probably caused by sensory input.
